# Inflammatory Tonsillar Disease and Its Cure

**Published:** 1914-10

**Authors:** Chester C. Cott

**Affiliations:** Buffalo


### Inflammatory Tonsillar Disease and Its Cure

CHESTER C. COTT, Buffalo.

The tonsils are composed of lymphoid tissue surrounded by a capsule. They lie in a fossa composed of the palato-glossus muscle anteriorly, palato-pharyngeus posteriorly and externally the superior constrictor muscle. The median surface of the tonsil which faces the mouth is covered by mucous membrane, and has from eight to twenty crypts which often extend the entire depth of the tonsil. The tonsil receives lymphatics from the nose. Those lymphatics which start at the tonsil go through the deep lymphatic chain under the sterno-cleido-mastoid muscle to the thoracic glands and then into the thoracic duct. The blood supply is from a branch of the facial or lingial or both.

Normal tonsils are able to absorb and kill bacteria, but if they are diseased they are positive sources of danger, as the bacteria then enter the crypts and tonsils and some proceed to the lymphatics of the neck and bronchial glands without hindrance, causing pulmonary tuberculosis, acute endocarditis, acute nephritis, acute articular rheumatism, etc. The tonsils are easily infected because they have so many deep crypts with poor drainage, some in fact draining upward into the supra-tonsilar fossa. Besides this fact that a diseased tonsil is continually becoming inflamed causing characteristic severe general reaction it at any moment may be the cause of one of the above mentioned internal diseases.

All inflammatory tonsillar diseases are caused by infection through the crypts. In one or more of them there is a focus which allows the bacteria to multiply, the epithelium breaks down, the germs advance and there is an acute follicular attack. This disease is sudden in onset and short in duration but may become chronic. When acute the follicles are full of dead epithelium, germs and leucocytes, which give a white color to their mouths. The rest of the tonsil is inflamed and swollen. The disease is very toxic causing severe headache and backache. The pain on swallowing permits only liquid food. Silver nitrate 10% sol. applied into each follicle as deeply as possible soon cures the attack, if accompanied with stern eliminative measures. If treatment does not soon cure the attack, the symptoms at least become more mild. Then the chronic form appears with mild but persistent symptoms. This may continue for a long time in spite of treatment. Sometimes the debris in the follicles becomes hardened and often quite large forming what are called tonsillar calculi.

During the course of an acute or chronic follicular tonsilitis one of the follicles, usually one which drains upward into the supratonsillar fossa, becomes plugged up and an abscess forms. This often starts first in the tonsil itself but soon localizes itself in the supratonsillar fossa. Then we have a supratonsillar abscess or quinsy sore throat. As the abscess enlarges the pain increases, the breath becomes very foul and there is inability to open the mouth as such action compresses the abscess and increases the pain. The soft palate covering the offending tonsil will be seen to bulge anteriorly more or less. The symptoms increase in severity until the abscess breaks or is opened. If spontaneous rupture takes place it is usually through the anterior pillar. If a physician is called he must open the abscess either through the anterior pillar half-way between the last molar tooth and the root of the uvula or behind the pillar. above the tonsil. When the abscess cavity is drained recovery is rapid, but the attack will almost certainly recur.

A tonsil that is subject to chronic inflammation becomes hypertrophied. Occasionally enlarged tonsils are seen in the new-born but as a rule the cause of enlargement is inflammation. In the young all the cells increase in number but only the connective tissue cells in those over twelve. Therefore the older the patient the firmer the tonsil. The chronic inflammation in the crypts besides causing hypertrophy sometimes results in hyperkeratosis or mycosis. In the former the epithelium of the crypts grows faster and becomes horny and projects from the mouth of the crypt. They are plainly visible as while spots. Often some other part of the mouth or throat is also affected. According to Ballenger the disease is self-limited, stopping in two or three years.

Mycosis of the tonsils is a mere incident in chronic tonsilitis. The fungi grow in the tonsils causing projections similar to those of hyperkeratosis. It does not succumb to treatment. A few follicles may be enlarged and cauterized at each sitting but the process is slow and uncertain. The only way positively to cure any and all the diseases I mentioned is to enucleate the tonsils. And when I say cure I mean cure to stay cured.

At this point permit me to say a few words on the function of the tonsil. The tonsil is simple lymphoid tissue confined in a capsule and as such would seem to be a simple lymph node. Its removal if this were so would cause no great harm. However there are those who claim that the tonsil has some great though unknown function, perhaps great because unknown. Dr. Gustave P. Head of the Chicago Post-Graduate Medical School says, "O. Levinstein of Berlin who is associated with Fraenkel in the Berlin Policlinic has done as much work and spent as much thought on the question of the tonsils as anyone. He says that all theories fall short of expressing what the function of the tonsils is. He leaves you entirely in a way without a leg to stand upon, finding evidence which seems to contradict that anyone of these theories explains the sole function of the tonsils. In this last he is probably right as no one single thing explains what the tonsil does. It may do all of them but one can safely assume that it is absolutely in every sense and in all senses a lymph node and as such should be protected from rude destruction. Whatever its humble office may be, it had better be allowed to perform it.''

Sir Jonathan Wright in the N. Y. Med. Jour., August 8, 1909, wrote that since a tonsil increased in size after a piece was cut off—it was a vital part of the body. This action was similar to the regeneration of certain kinds of star fish, who may have a part amputated by disease. This part subsequently will regenerate. He concludes that the knowledge derived from all these sources and the experimental evidence unites in pointing to the function of the tonsils as one of defence against infection. The tendency to enlargement seems a persistence of the process by which the function was evolved.

In reviewing this article, Dr. Head as Editor of the 1909 Year Book of Eye, Ear, Nose and Throat, says: ''It seems to the editor that too much stress is laid upon the evolutionary idea in dealing with modern civilized man. Nature takes a long time (we are accustomed to saying ages) to produce evolutionary changes and eliminate those individuals which according to her standards are unfit to survive. Nature cares nothing for the individual, little even for the race in her merciless selection and elimination. . . . When we see what nature is doing by means of the vermiform appendix, the three tonsils, cancer, nervous diseases, etc., it is hard to believe she is not using every possible means to eliminate a large share of civilized beings from her scheme of life. Man has by his reason developed the unnatural conditions of modern life and unless by the use of his intellectual powers he can devise means of saving the individual from his physical sins there will soon be no individuals to save. Doubtless if nature is given sway, in the course of ages she would produce a race which would not suffer appendicitis by killing off all those with improper appendices, but the surgeon steps in and thwarts her intentions by cutting out the appendix and saving the individual as fast as nature dooms him. Another weak point is the tonsillar ring and the ''good'' mother is wreaking her vengeance in no uncertain manner at this point. Here however modern civilization has developed her throat surgeons whose business it is to halt destruction as it shows its ruthless hand on the innocent child and says in effect, ''I will interfere with the suffering and untold damage inflicted on the individual by the lymphatics of the throat by cutting them out.'' Of course nature will find other weak points and her destroying power will triumph in the end unless civilized man is able to wrest enough more of her secrets to thwart her as he is now doing on the organs mentioned. . . . A large part of the practice of medicine is built upon the theory of saving man from the punishment which nature is bent on inflicting on him for his physical sins and there is no call to hesitate in the matter of the tonsils any more than in the matter of the appendix or cancer. The physician shapes his actions on the plan of helping the individual by helping him to secure the greatest amount possible of nature's beneficence and avoiding the greatest amount possible of her malevolence."

After this digression let us consider the treatment of tonsillar diseases. There are three methods: internal medication, local applications and surgical interference. The acute inflammatory diseases may be cured by the first two methods. Quinsy sore throat will not be cured until the abscess breaks or is opened when it will subside until another attack. The chronic affectations of the tonsils require surgical interference to establish a cure. When anything short of this is done alleviation of the symptoms may be temporarily obtained but a recurrence is almost certain.

What operation is the next question? The only one we do now is enucleation of the tonsils. This absolutely cures all tonsillar affections. If a tonsil is not perfectly enucleated trouble may still ensue because the follicles which are always diseased run as deep as the outer surface of the tonsils in some cases. So if any tissue is left in the tonsillar fossa it is very likely diseased tissue.

We employ three methods for enucleation. Sluder's method according to the originator can be used in all cases. One uses only the Sluder tonsilotome. This instrument is powerfully built because quite a bit of pressure must be used. The blade is dull. The tonsil is approached from the opposite side, its lower end engaged in the opening and then the tonsil is pushed forward and outward until it touches the prominence on the median surface of the mandible caused by the last molar tooth. Now the convex bed of the tonsil is pushed against the convex surface of this prominence. This action forces the tonsil through the tonsilotome. When the entire tonsil is through, the blade of the instrument is pushed down, securely locking the tonsil in the instrument. Now the anterior pillar is palpated to be sure the entire tonsil is secured. If necessary any remaining part is forced through the guillotine with the index finger. Then the blade is pushed down as hard as possible. If the capsule does not separate easily from its bed, the index finger loosens it. This method is practically bloodless and gives perfect results in skillful hands.

The operation which I prefer is Beck's modification of

Sluder's method. According to Dr. Beck this method can be used in all children and in 70% of adults. Beck's snare is used. The lower end of the tonsil is engaged in the ring and the tonsil pulled forward and upward and outward. This action pushes the tonsil against the anterior pillar. The index finger of the opposite hand then forces the tonsil through the ring and is then held there until the snare wire is drawn tight. The finger is then taken off of the anterior pillar and the tonsil, held in the snare, is inspected and grasped in a tonsil forceps. Then the capsule is separated from its bed by tightening the snare wire. Beck's method gives perfect results also. To me it is much simpler than the Sluder method and injury to the pillars of the tonsil is impossible.

The third method is the dissection and snare method. The tonsil is grasped by a tonsil forceps, pulled toward the median line and dissected from its bed by a curved-bladed dull-pointed scissors. The band holding the tonsil to the anterior pillar usually must be cut but most of the dissecting can be done by inserting the scissors closed and opening the blades in the tissues, thus separating the tonsil in its capsule from the surrounding muscles. Occasionally the entire border of the tonsil must be cut away from the pillars, before blunt dissection can proceed. Of course there is more hemorrhage with this method. However, there is no tonsil which cannot be enucleated after this manner. It will take care of all tonsils which cannot be removed by the other methods, and without injury to the pillars.

The object of this paper is to show you that although acute diseases of the tonsil may be cured of the attacks, a recurrence is almost certain because of the structure of the tonsil. To protect the body from general diseases caused by germs which enter through the diseased tonsils and to prevent absolutely recurrent attacks of tonsilitis, enucleation is necessary. The tonsil is a simple lymph node and as such when removed is not missed in the body functions. Instead a port of entry of bacteria is closed. Its complete removal is accomplished by several methods, of which I prefer Beck's method when it can be used, and the Sluder or the dissection methods in all other cases.

